# Associations between nasopharyngeal carriage of Group B Streptococcus and other respiratory pathogens during early infancy

**DOI:** 10.1186/s12866-016-0714-7

**Published:** 2016-05-27

**Authors:** Ebenezer Foster-Nyarko, Brenda Kwambana, Odutola Aderonke, Fatima Ceesay, Sheikh Jarju, Abdoulie Bojang, Jessica McLellan, James Jafali, Beate Kampmann, Martin O. Ota, Ifedayo Adetifa, Martin Antonio

**Affiliations:** Vaccines and Immunity Theme, Medical Research Council Unit, Banjul, The Gambia; Current Address: WHO Regional Office for Africa, Brazzaville, Congo; Disease Control and Elimination Theme, Medical Research Council Unit, Banjul, The Gambia; Current Address: Infectious Diseases Epidemiology, London School of Hygiene & Tropical Medicine, London, UK; Faculty of Infectious and Tropical Diseases, London School of Hygiene & Tropical Medicine, London, UK; Microbiology and Infection Unit, Warwick Medical School, University of Warwick, Coventry, UK

**Keywords:** Nasopharyngeal, Group B streptococcus, Beta-haemolytic streptococci, Gambia

## Abstract

**Background:**

In West Africa, the carriage of Group B Streptococcus (GBS), among infants is poorly characterised. We investigated co-carriage of GBS with other respiratory pathogens in the infants’ nasopharynx in The Gambia.

**Methods:**

We assessed the carriage, serotypes and antibiotic susceptibility of Beta-haemolytic Streptococci (BHS) groups A-G; along with the carriage of *Streptococcus pneumoniae; Haemophilus influenzae; Staphylococcus aureus* and *Moraxella catarrhalis* in 1200 two-month old infants.

**Results:**

The BHS prevalence was 20.0 % and GBS dominated (13.8 %), particularly serotypes V and II; serotype V being negatively associated with *H. Influenzae* carriage (OR 0.41 [95 % CI: 0.18–0.93], *p* = 0.033). Although co-colonization of GBS and other BHS was not seen, colonization with GBS was positively associated with *S. aureus* (OR 1.89 [95 % CI: 1.33–2.69], *P* < 0.001) and negatively associated with *S. pneumoniae* (OR 0.47 [95 % CI: 0.33–0.67], p < 0.001) and *M. catarrhalis* (OR 0.61 [95 % CI: 0.40–0.92], *p* = 0.017). ≥ 89 % of GBS isolates were susceptible to most antibiotics tested, except for tetracycline resistance, which was 89 %.

**Conclusion:**

This study provides baseline data on the carriage of GBS in two month old infants from West Africa. The dominant serotypes of GBS in this setting are serotypes V and II. This may be important for future GBS vaccine development for the West African sub-region.

## Background

The beta-haemolytic group of Streptococci (BHS) causes a wide variety of infections ranging from mild skin infections to life threatening diseases including sepsis and meningitis [1, 2]. BHS are classified into Lancefield serogroups (A –G) depending on their cell wall structure [3]. BHS are amongst the top ten causes of invasive bacterial diseases in adults and children beyond the neonatal period [4, 5].

A sub-group of the BHS is Group B Streptococcus (GBS) also known as *Streptococcus agalactiae,* which is a major cause of neonatal sepsis and meningitis with significant morbidity and mortality rates in the developed world [6, 7]. However, there are historical and now emerging data suggesting that GBS may also be a frequent cause of neonatal sepsis in sub-Saharan Africa [8–10]. GBS comprises ten serotypes (Ia, Ib, II-IX) and there are geographical variations with respect to the predominant serotypes in invasive disease [7, 11, 12].

Microbes colonizing the nasopharynx can invade the blood stream or migrate to contiguous surfaces of the respiratory tract [13]. Consequently, nasopharyngeal carriage of respiratory pathogens such as *S. pneumoniae* is a risk factor for the development of several infections including pneumonia, meningitis and sepsis [14]. Droplet secretions from the nasopharynx are also an important mechanism for the horizontal transfer of pathogens [15]. The epidemiology, transmission and nasopharyngeal carriage of *S. pneumoniae and Haemophilus influenzae* type b (Hib) have been studied in the Gambia [16, 17]. However, nasopharyngeal co-carriage of GBS with other respiratory pathogens in the post pneumococcal and Hib conjugate vaccine era has not been described in West Africa.

This study investigated co-carriage of GBS with other BHS and four common respiratory pathogens colonizing the nasopharyngeal mucosae beyond the first week of life. We also characterised the carriage, serotypes and antibiotic susceptibility of GBS in these Gambian infants who had not yet been vaccinated with the pneumococcal conjugate and Hib vaccines. This study provides critical baseline data on the carriage of GBS and other BHS in the sub-region.

## Methods

### Study area and population

The study was conducted in the Fajikunda district, a typical peri-urban setting in the Western region of The Gambia. As described previously, this area is representative of similar communities situated near large cities in West Africa [18].

Nasopharyngeal swabs (NPS) were obtained between July 2011 and May 2012 from 1200 healthy infants aged two months of consenting parents at the Fajikunda infants’ well-being clinic.

The Gambia Government/MRC Joint Ethics Committee approved the study. Trained field workers/nurses explained the contents of the study information sheet to parents/guardians in their own language. Parents/guardians of all study participants gave written informed consent prior to enrolment.

The study team administered questionnaires to record history of acute respiratory tract infection, place of birth (whether in a health facility or at home), cooking method, ethnic group, number of other infants less than 5 years in the household and sex.

### Nasopharyngeal swab sampling methods

NPS were collected as described previously [18] and were processed for the isolation of BHS and four additional respiratory pathogens including *S. pneumoniae, H. influenzae, S. aureus and M. catarrhalis*.

### Microbiological methods

#### Identification and isolation of bacterial pathogens

The broth enrichment method for enhancing the yield of *S. pneumoniae* [19] was adapted to improve the yield of BHS and other respiratory pathogens. Briefly, NPS stored in Skim-milk Tryptone Glucose Glycerol were thawed at room temperature (25 °C) and briefly vortexed. 200 μl of the NPS in Skim-milk Tryptone Glucose Glycerol was inoculated into 5mls of Todd-Hewitt Broth with 5 % Yeast Extract (Oxoid, Basingstoke, UK) containing 1 ml of rabbit serum (B&K Universal Ltd, Grimston, East Yorkshire, UK) and incubated for 5-6 h at 37 °C in ambient air.

For BHS isolation, 100 μl aliquots were streaked onto a 5 % Sheep Blood Agar plate containing a 1:50000 dilution of crystal violet to inhibit Staphylococcus growth*.* For *M. catarrhalis, H. influenzae, S. pneumoniae* and *S. aureus* isolation, aliquots of 100 μl were streaked onto each of Chocolate agar, Bacitracin chocolate agar, Gentamicin sheep blood agar and Mannitol salt agar plates, respectively. All plates were incubated overnight at 37 °C; Gentamicin sheep blood agar, Bacitracin chocolate agar and Chocolate agar plates in 5 % CO_2_; Crystal violet blood agar and Mannitol Salt agar in ambient air.

Isolation and identification of bacterial pathogens (*H. influenzae, S. pneumoniae, M. catarrhalis* and *S. aureus*) were carried out as previously reported [18]. For *H. influenzae;* type B specific antisera obtained from Statens Serum Institut (SSI, Copenhagen, Denmark) was used to serotype the isolates as Hib or non-Hib.

Isolation of BHS was carried out as follows. Following overnight incubation of the Crystal violet blood agar plates, 2-3 colourless or grey, dry or moist, beta-haemolytic colonies were individually streaked to Blood agar plates. A 0.04 Units bacitracin disk (BD Oxoid, Basingstoke, UK) was placed in the first quadrant for the preliminary identification of Group A Streptococcus (GAS). The plates were then incubated overnight at 37 °C in 5 % CO_2_. All bacitracin negative, catalase negative colonies were subjected to the Christie, Atkins, Munch and Peterson test for the preliminary identification of GBS. *S. agalactiae* (ATCC 12386) were inoculated as negative and positive controls, respectively, for each batch of testing. All beta-haemolytic isolates (including all isolates preliminarily identified as GAS and GBS) were further grouped using the Streptex® Streptococcal grouping kit (Remel & Oxoid, Thermo Fisher Scientific, Basingstoke, UK) for confirmation as GAS, GBS, Group C (GCS) or group G (GGS). All isolates were stored in 15 % Glycerol broth at −70 °C until further testing.

### Serotyping of GBS isolates

#### Latex agglutination method

One hundred thirty-three out of 162 (82 %) GBS isolates were successfully revived for serotyping by the slide latex agglutination method. Confirmed GBS isolates were streaked onto a 5 % Blood agar plate to obtain fresh cultures. Serotyping was carried out using Strep-B-latex antisera® (SSI, Copenhagen, Denmark) as per manufacturer’s instructions. For each batch of serotyping, serotype specific GBS controls strains Ia, Ib, II – IX obtained from Centres for Disease Control and Prevention, Atlanta (Strain numbers 2008232728, 2008232729, 2008232738, 2008232582, 2011201884, 2008232731, 2010228816, 4832–06, 5030–08, 7509–07 respectively) [20] were used as positive controls.

#### Conventional PCR serotyping method

All the GBS strains which were non-typeable (NT) by the latex agglutination method (21/133, 15.8 %) were subjected to the conventional serotyping method (Polymerase Chain Reaction, PCR). In addition, 15.0 % of all the GBS strains typed by the latex agglutination method (20/133) were confirmed by PCR.

DNA was extracted from pure overnight colonies of GBS following as per the World Health Organization (WHO) Laboratory manual for the diagnosis of meningitis [21].

Serotyping was then carried out using the method of Imperi et al. [20]. For each run, non-template negative controls as well as positive GBS control strains (serotypes Ia, Ib, II-IX (Centres for Disease Control and Prevention, Atlanta, USA) were included.

#### Susceptibility testing

All the 133 GBS isolates that were serotyped were further tested for their susceptibility to Penicillin (10 units), Chloramphenicol (30 μg), Tetracycline (30 μg) and Erythromycin (15 μg) discs (BD Oxoid, Basingstoke, United Kingdom) by the Kirby-Bauer method. E-tests (Biomerieux, Basingstoke, United Kingdom Limited) were performed for all isolates showing resistant or intermediate sensitivities by the Kirby-Bauer method.

All interpretation of antibiotic susceptibilities were done using CLSI breakpoints [22].

All reagents and the disc dispenser were obtained from BD Oxoid, Basingstoke, UK, except otherwise stated*.*

The MRC Unit The Gambia, molecular microbiology laboratory submits to the external quality assurance programme of the UK National External Quality Assessment Service [23] and is a World Health Organization (WHO) Regional Reference Laboratory for invasive bacterial pathogens.

#### Statistical analysis

Data management was done using SQL integrated database with a Microsoft Access front-end linked to field, clinical and laboratory databases.

All statistical analyses were conducted in Stata version 12 for Windows (Stata Corp, College Station, TX). Any association with a p value of <0.05 was considered to be significant.

Categorical variables were summarised using frequency counts and percentages, n (%). Mean (SD) and median (IQR) values were used to describe normally and non-normally distributed continuous variables, respectively.

Cross tabulations (with Fishers’ exact tests for associations) were used to describe and compare the prevalence of BHS carriage across different levels of individual potential risk factors including carriage of other pathogens (categorical variables). Distributions of continuous variables were compared between BHS carriers and non-carriers using independent sample t-test (if approximately normal) or non-parametric Wilcoxon rank-sum test (if non-normal).

Univariable (simple) and multivariable (multiple) logistic regression analyses were then performed to estimate unadjusted and adjusted odds ratios (respectively) with their 95 % confidence intervals to quantify the associations of BHS carriage prevalence with the potential risk factors. The confounding effects of other variables and presence of statistical interactions were also assessed during multiple logistic regression analyses. The combination of risk factors mutually associated with BHS was identified using a stepwise backward elimination (logistic regression) procedure, with an exclusion *p* value of 0.1.

## Results

**Fig. 1:**
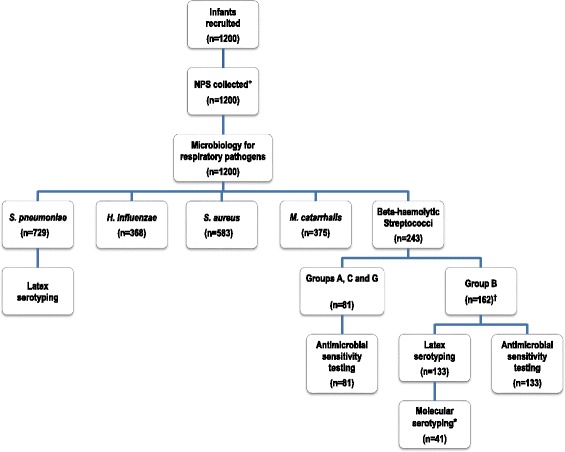
Algorithm for recruitment and sample processing flow. ^+^ 1200 infants aged 2 months of consenting mothers were recruited for the study; however 1170 (97.5 %) had complete data for analysis. ^†^ 162 GBS isolates were recovered in the study, however 133 (82 %) were successfully revived for serotyping. * 21 GBS isolates were non-typeable (NT) by the latex agglutination method and were subjected to the conventional Polymerase Chain Reaction serotyping method. In addition, 20 isolates typed by the latex agglutination method (15 %) were confirmed by PCR

There were 1200 participants aged 2 months of which 1170 (97.5 %) had complete data for analysis. The algorithm for recruitment and sample processing flow is shown in Fig. 1. The characteristics of the study population are summarized in Table 1.

**Table 1:** Demographic characteristic of 1170 Gambian 2-month old infants participants

Characteristic	n (%)	%
Sex		
Male	579	49.5
Female	591	50.5
Place of birth		
Other^b^	98	8.4
Health Facility	1072	91.6
Sleep Rm^a^		
0	374	32.4
1	433	37.5
2	214	18.5
3^b^	134	11.6
History of previous antibiotic use		
No	1170	100
Yes	0	0
History of ARI		
No	1041	89.0
Yes	129	11.0

*ARI* acute respiratory infection; ^a^Number of other siblings <5 years sleeping in the same room with subject (data available for 1155 infants); ^b^Other, At home/delivered by a traditional birth attendant

### Prevalence of bacterial species

**Fig. 2:**
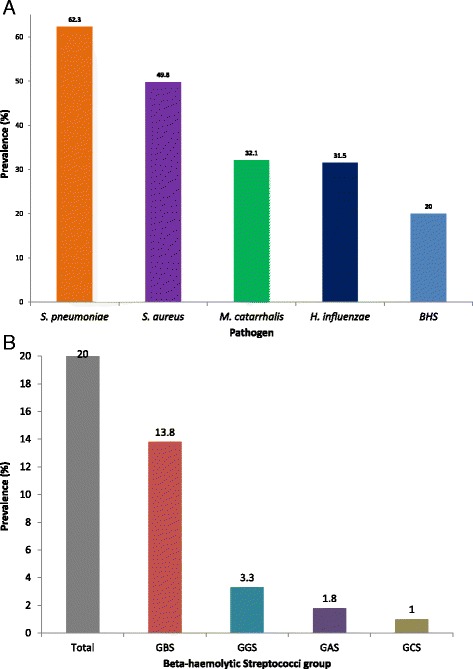
**a** Nasopharyngeal carriage of respiratory pathogens in 2-month old Gambian infants. **b** Nasopharyngeal carriage of Beta-haemolytic Streptococci In 2-month old Gambian infants. BHS – Beta-haemolytic Streptococci; GBS – Group B Streptococci; GGS – Group G; Streptococci; GAS – Group A Streptococci; GCS – Group C Streptococci

Figure 2a shows carriage of BHS and 4 common respiratory pathogens in the nasopharynx; with *S. pneumoniae* being the highest (729/1170, 62.3 %) and *H. influenzae* being the least (368/1170, 31.5 %) in carriage respectively. All the *H. influenzae* isolates recovered were serotyped to be non-Hib.

Of 234 out of 1170 (20.0 %) BHS isolated, GBS was the most dominant (162/234, 69.2 %), and GCS the least prevalent (12/234; 5.1 %) (Fig. 2b). Two isolates that could not be assigned to any Lancefield group (BHS non-typeable) were excluded from the overall prevalence and risk factor analyses. Similar proportions of males and females carried BHS in their nasopharynx (127/579 (21.9 %) and 107/591 (18.1 %) respectively).

### Serotype distribution of GBS

Of the 162 GBS isolates that were recovered, 133 (≈82 %) were successfully revived for serotyping.

Nine different GBS serotypes were identified of which the most prevalent were serotypes V (50/133, 37.6 %) and II (46/133, 34.6 %), which together constituted approximately 72 % of all the GBS serotypes identified. The other GBS serotypes were Ia (10/133, 7.5 %), IV (8/133, 6.0 %), Ib (7/133, 5.3 %), VII (5/133, 3.8 %), VIII (1/133, 0.8 %), III (1/133, 0.8 %) and VI (1/133, 0.8 %) (Table 2). Serotyping by PCR resolved all strains that were non-typeable by latex agglutination, except for 2 isolates (2/133, 1.5 %). Two infants were colonized with two different serotypes (V & VII, and V & VIII respectively).

**Table 2:** Temporal changes in the Group B Streptococcus Serotype distribution from the same geographic region (Fajikunda) in The Gambia: 20 years apart

Study	Year	Description	Total number of isolates	% with serotype								
				Ia	II	III	IV	V	VI	VII	VIII	NT
Ref 24	May 1992 to February 1993	Maternal and infant colonization	32^a^	19	28	6	3	38	-	- ^b^	- ^b^	6
Present study	July 2011 and May 2012	Infant colonization	133	7.5	34.6	0.8	6.0	37.6	0.8	3.8	0.8	1.5

^a^Lancefield grouping was performed for only serotypes I-VI and did not include serotypes VI-IX

The PCR method was in agreement with the latex agglutination method in the 15.0 % (20 isolates) that were confirmed by PCR.

### Risk factors for BHS carriage

In univariate and multivariable logistic regression analyses of risk factors for BHS carriage, carriage of the Streptococcus groups A-G was not associated with gender, ethnicity, home or hospital delivery, or number of other siblings <5 years sleeping in the same room as the participating infant. There were no significant associations between the carriage of GAS or GCS and a history of acute respiratory infection. In contrast, a history of acute respiratory infection was significantly associated with carriage of GGS (OR 2.42, [95 % CI: 1.12–5.23], *p* = 0.025).

As shown in Table 3, GBS carriage was negatively associated with a history of acute respiratory infection (OR 0.28, [95 % CI: 0.13–0.63], *p* = 0.002).

**Table 3:** Risk factors for carriage of Group B Streptococcus

Risk factor	Category	N	Positivity of outcome	Univariable analysis		Multivariable analysis	
			n (%)	OR (95 % CI)	p value	OR (95 % CI)	*p* value
Sex	Male	579	90 (15.54)	1		1	
	Female	591	72 (12.18)	0.75 (0.54;1.05)	0.097	0.73 (0.52;1.04)	0.08
Place of Birth	Other	98	9 (9.18)	1			
	H/F	1072	153 (14.27)	1.65 (0.81;3.34)	0.167	-	-
Tribe	Mandika	463	62 (13.39)	1			
	Wolof	164	22 (13.41)	1 (0.59;1.69)	0.994	-	-
	Fula	144	19 (13.19)	0.98 (0.57;1.71)	0.952	-	-
	Jola	215	24 (11.16)	0.81 (0.49;1.34)	0.418	-	-
	Others	184	35 (19.02)	1.52 (0.96;2.39)	0.072	-	-
*sleep Room	0	374	59 (15.78)	1			
	1	433	58 (13.39)	0.83 (0.56;1.22)	0.339	-	-
	2	214	28 (13.08)	0.8 (0.49;1.31)	0.377	-	-
	3+	134	14 (10.45)	0.62 (0.34;1.16)	0.134	-	-
History of ARI	0	1041	155 (14.89)	1		1	
	1	129	7 (5.43)	0.33 (0.15;0.72)	0.005	0.28 (0.13;0.63)	0.002
*S. pneumoniae*	Negative	441	88 (19.95)	1		1	
	Positive	729	74 (10.15)	0.45 (0.32;0.63)	<0.001	0.47 (0.33;0.67)	<0.001
*H. influenzae*	Negative	802	124 (15.46)	1			
	Positive	368	38 (10.33)	0.63 (0.43;0.93)	0.019	-	-
*S. aureus*	Negative	587	58 (9.88)	1		1	
	Positive	583	104 (17.84)	1.98 (1.4;2.79)	<0.001	1.89 (1.33;2.69)	<0.001
*M. catarrhalis*	Negative	795	126 (15.85)	1		1	
	Positive	375	36 (9.6)	0.56 (0.38;0.84)	0.004	0.61 (0.40;0.92)	0.017

*H/F* health facility, *ARI* acute respiratory infection; *Number of other siblings <5 years sleeping in the same room with subject. (−) indicates variables which were not included in the multivariate analysis

### Co-carriage of GBS and other pathogens

GBS was not carried along with any other BHS in our study population. GBS co-colonization was most frequent with *S. aureus* (104/583, 17.8 %), followed by *H. influenzae* (38/368, 10.3 %), then *S. pneumoniae* (74/729, 10.2 %), and *M. catarrhalis* (36/375, 9.6 %) (Table 3).

### Association between BHS and four common respiratory pathogens

As shown in Table 3, carriage of GBS was negatively associated with that of *S. pneumoniae* and *M. catarrhalis but* positively associated with carriage of *S. aureus* (OR 1.89 [95 % CI: 1.33–2.69], *p* < 0.001). Although carriage of GBS was also negatively associated with that of *H. influenzae* in the univariate analysis this association was not confirmed in multivariable analysis. We also found a significant negative association between GBS serotype V and the carriage of *H. influenzae* (OR 0.41 [95 % CI: 0.18 –0.93], *p* = 0.033).

### Antibiotic susceptibility of GBS

All the GBS isolates tested were susceptible to Penicillin and 97.0 % (129/133) were susceptible to Chloramphenicol. Resistance was highest to Tetracycline (119/133, 89.5 %) with a minority of isolates resistant to Erythromycin (1/133, 0.8 %).

## Discussion

To our knowledge, this is the first report of co-carriage of GBS with other respiratory pathogens in the nasopharynx of two-month old infants. These data also provide an update on the circulating serotypes of GBS, as available data from West Africa are from the 1990’s [24]. Additionally, we describe for the first time, the nasopharyngeal prevalence of other GBS and BHS beyond neonatal period from West Africa.

We found a prevalence of GBS in the nasopharynx 14.0 % (162/1170), with an overall prevalence of BHS of 20.0 % (234/1170) in NPS examined (Fig. 2a and b).

Although data on nasopharyngeal carriage of GBS during early infancy is sparse in West Africa, our findings are consistent with an earlier study in the Gambia [24], albeit in infants less than 24 h old, and from different body sites.

The 13-valent pneumococcal conjugate vaccine (PCV-13) has been in use in The Gambia since April 2011, before the start of this study. Few studies have reported changes in the epidemiology of bacterial carriage and diseases associated with widespread use of the pneumococcal conjugate vaccine [25, 26]. However, the prevalence of GBS carriage and disease in the post-PCV era has not been properly described. New pneumococcal vaccines capable of inducing non-serotype dependent and broadly reacting immunity have been developed and are in early clinical trials [27]. Introduction of these new vaccines with the potential to disrupt nasopharyngeal ecology may be associated with the potential risk of species replacement including a more prominent role for related organisms such as *S. aureus*.

We previously showed that *S. pneumoniae* was positively associated with both *H. influenzae* and *M. catarrhalis*, and negatively associated with *S. aureus* in NP carriage [18]. In this study, we report for the first time, an interesting interplay of associations between GBS and other commonly studied respiratory pathogens. The negative association we found between GBS and *S. pneumoniae* and the positive association with *S. aureu*s suggest that GBS may occupy a niche that overlaps with that of *S. pneumoniae* and *S. aureus.* It is interesting to note that the niche overlap between these pathogens may actually be quite large amongst infants from this setting as *S. pneumoniae* and *S. aureus* have both also been isolated from the oropharynx where GBS typically resides [18]. With respect to *S. pneumoniae*, the co-existence appears to be antagonistic, whereas the converse is true for *S. aureus.* Furthermore, no GBS isolate was co-carried with another BHS. This is an interesting finding and may suggest an exclusive pattern of co-colonization amongst BHS of different Lancefield groups, probably due to their metabolic similarities. With the development of pneumococcal protein vaccines that may wipe out carriage of *S. pneumoniae,* there’s a potential risk of increase in GBS carriage with possible clinical consequences.

Although data on carriage of non-BHS in infants belonging to this age group is sparse, our findings are consistent with the existing reports from other parts of the world [28, 29]. In a cross-sectional study such as ours, it is possible to have missed any BHS which may have a seasonal pattern of colonization, such as have been shown with *S. pneumonia* serotypes, 1 and 5 [30]. Confirming this would require studying a larger cohort of infants in future studies to investigate BHS carriage longitudinally.

In this study, the predominant GBS serotypes were V (37.6 %) similar to what was found in an earlier study in The Gambia, and II (34.6 %). In contrast to our findings, this other study conducted two decades ago did not find appreciable numbers of serotype II [24]. This suggests the existence of temporal changes in the carriage of GBS and most likely other BHS serotypes. The prevalence also varies geographically. Globally serotype III plays a major role in both early-onset and late-onset disease as well as carriage in developing and developed countries [31, 32]. However, this serotype comprised <1 % of GBS isolates in our study. There is no GBS vaccine available currently, although some vaccine candidates have been tested in preclinical studies [33]. A Novartis glycoconjugate vaccine containing serotypes Ia, Ib and III is currently undergoing phase II trial [34]. Although the carriage of GBS has not been shown to predispose to invasive disease unlike in organisms such as *S. pneumoniae*, it may be worthwhile for future vaccine development targeting developing countries such as The Gambia to consider the geographical differences in serotype distribution.

Latex agglutination is the conventional method for serotyping. Our data demonstrates the added utility of PCR serotyping as it resolved majority of the serotypes (19 of 21) identified as non-typeable by latex agglutination. The usefulness of such molecular serotyping techniques has been reported in the literature [35].

Penicillin is the recommended first-line of treatment for streptococcal infections, including GBS infections and all GBS isolates in this study were found to be Penicillin susceptible. Similar to other regions, we observed a high rate of tetracycline resistance [36]; however this antibiotic is not commonly used in children. Macrolides are the recommended second line treatment option. Globally, there is growing evidence of emerging resistance of GBS to erythromycin [37, 38], making it important to probe resistance patterns to this antibiotic. In our study, <1 % of GBS isolates were resistant to erythromycin; with approximately 11 % showing intermediate susceptibility; an indication that macrolide antimicrobial resistance is not a major concern in our setting at this time. Our findings are similar to reports from Brazil where 4.7 % of colonizing GBS isolates were resistant to erythromycin [39]; and Australia where 6.4 % of GBS isolates were resistant to erythromycin [40].

Host specific factors such as age, race, breastfeeding, attendance at day care amongst others have been shown to affect colonization of *S. pneumoniae*, *S. aureus*, *M. catarrhalis*, and *H. influenzae* [41]. Intercurrent or past episodes of respiratory tract infections are also known to increase colonization by these pathogens [41].

Contrary to results from other studies, multiple logistic regression analyses in this study did not yield any association between GBS carriage and gender, ethnic group and overcrowding. In addition, GBS carriage was inversely associated with a history of acute respiratory infection. This finding is interesting, as it suggests that recent acute respiratory infection may have an impact on GBS carriage. However, we note that none of the study participants had any history of previous antibiotic use. Thus it may likely be a chance finding (although statistically significant).

## Conclusions

In light of our findings, and evidence from elsewhere [25, 26], we recommend that invasive bacterial disease surveillance following introduction of new vaccines should include monitoring not just for serotype replacement but also species replacement in carriage and invasive pneumococcal disease. In addition, close surveillance monitoring is required to detect the emergence of bacterial antimicrobial resistance in the Gambia. We have shown that GBS has negative and positive associations with *S. pneumoniae* and *S. aureus* respectively. We have also shown that the GBS serotype V remains dominant in colonization of Gambian infants, and is negatively associated with the carriage *of H. influenzae*. We have also found an emergence of GBS serotype II. Although preliminary, this information is important information for future vaccine development for West African countries.

## Abbreviations

BHS, beta-haemolytic streptococci; GAS, Group A streptococcus; GBS, Group B streptococcus; GCS, Group C streptococcus; GDS, Group D streptococcus; GES, Group E streptococcus; GFS, Group F streptococcus; GGS, Group G streptococcus; Hib, *Haemophilus influenzae* type B; NT, non-typeable; NPS, nasopharyngeal swabs; PCV, pneumococcal conjugate vaccine
